# Effect of daily stressors and collective efficacy on post-traumatic stress symptoms among internally displaced persons in post-war northern Sri Lanka

**DOI:** 10.1192/bjo.2023.563

**Published:** 2023-10-11

**Authors:** Daya Somasundaram, Rohan Jayasuriya, Ruwanthi Perera, Umaharan Thamotharampillai, Rajitha Wickremasinghe, Alvin Kuowei Tay

**Affiliations:** Department of Psychiatry, University of Jaffna, Jaffna, Sri Lanka; Department of Public Health, Faculty of Medicine, University of Kelaniya, Kelaniya, Sri Lanka; Department of Rogavijnana, Faculty of Indigenous Medicine, Gampaha Wickramarachchi University of Indigenous Medicine, Yakkala, Sri Lanka; Discipline of Psychiatry and Mental Health, School of Clinical Medicine, University of New South Wales, Sydney, Australia

**Keywords:** War trauma, daily stressors, collective efficacy, Sri Lanka, moderation and mediation

## Abstract

**Background:**

Daily stressors have been shown to mediate the relationship of war trauma and trauma-related distress among refugees and internally displaced persons exposed to war and conflict.

**Aims:**

To examine the extent to which the relationship between war-related trauma and mental distress was mediated by daily stressors and collective efficacy among internally displaced communities a decade after exposure to war.

**Method:**

In a cross-sectional study, we recruited a random sample of residents in villages severely affected by conflict in five districts in the Northern Province of Sri Lanka. Measures of war trauma, daily stressors, collective efficacy and post-traumatic stress symptoms (PTSS) were examined. Statistical analyses of the mediating and moderating effects of daily stressors were conducted using regression based methods.

**Results:**

Daily stressors mediated the association of war trauma and PTSS, as both paths of the indirect effect, war trauma to daily stressors and daily stressors to PTSS, were significant. The predictive effect of war trauma on PTSS was positive and significant at moderate and high levels of daily stressors but not at low levels. Higher levels of neighbourhood informal social control, a component of collective efficacy, function as a protective factor to reduce effects of war trauma and daily stressors on mental distress in this population.

**Conclusions:**

Daily stressors are an important mediator in the well-established relationship between war exposure and traumatic stress among internally displaced persons, even a decade after the conflict. Mental health and psychosocial support programmes that aim to address mental distress among war-affected communities could reduce daily stressors and enhance collective efficacy in this context.

At the end of 2022, a record 108.4 million people worldwide were forcibly displaced due to conflict and violence.^1^ This includes 62.5 million internally displaced persons, forced sometimes multiple times to leave their communities but still living within the borders of their home country. Traumatised survivors of war or political violence often have complex mental health problems, with anxiety, depressive and cognitive disturbances.^2,3^ In such settings, social functioning has also been damaged. This is generally termed ‘tearing of the social fabric’ or ‘collective trauma’, as social networks, structures and neighbourhood cohesion have been damaged.^4,5^ Measures of social well-being such as social capital, trust, social cohesion and collective efficacy can be useful markers for collective trauma, and their decline indicators of social dysfunction.^6,7^ The ongoing psychosocial problems and current stressors hamper daily functioning and engagement in relationships and meaningful livelihood.

## ‘Daily stressors’ model

Miller & Rasmussen^8^ initially proposed the ‘daily stressors’ model to include the unrecognised influence of commonly occurring stressors in the immediate environment as predictors of high levels of mental distress among those exposed to war and conflict. Daily stressors have been identified as mediators in the relationship between exposure to war and mental distress in several studies. Evidence of this mediation has been found among Bhutanese refugees in Nepal,^9^ in Darfuri refugees in Eastern Chad^10^ and, more recently, among the Rohingya refugees displaced to Bangladesh.^11^ This model was also tested among internally displaced persons, Nepalese exposed to chronic civil conflict^12^ and youth in Sierra Leone.^13^

In subsequent conceptualising, Miller & Rasmussen differentiated daily stressors to consist of (a) lower-intensity daily stressors, (b) potentially traumatic daily stressors and (c) direct exposure to war-related violence and loss, as these affect mental health and psychosocial well-being.^14^ Subsequently, based on a socio-ecological framework, additional paths were suggested and it was postulated that the effects of war-related trauma exposure may be mediated or moderated by daily stressors arising from the conflict situation.^15^ However, in protracted conflict settings it is difficult to differentiate between the types of stressor as most ‘daily stressors’ relate to daily living difficulties that existed before the war and were worsened by deprivations during and after it, or were indirectly caused by effects of the war and or were due to resettlement. For example, low mood due to post-traumatic stress disorder (PTSD) or depression due to war trauma can lead to a vicious cycle of inactivity and withdrawal that results in unemployment and poverty that can worsen depression which, in turn, increases withdrawal and inactivity; a vicious cycle that can potentially be reversed by appropriate post-war recovery interventions.^16–18^ In addition, the data collections were usually contemporaneous with war and emergencies, or were only a few years after the cessation of hostilities,^8,9,11,12^ making it difficult to formulate and respond to questions that can distinguish different types of daily stressor. The examination of the daily stressors model when there is a long gap between the exposure to war trauma and current daily stressors in settings where the populations affected have been resettled may provide additional insight to make these differentiations and to test the mediation and moderation effects of daily stressors.

## Protective factors: social capital, social cohesion and collective efficacy

Research on the mental health of those exposed to natural disaster and war trauma have sought factors that buffer and are protective against mental distress. Such studies have investigated individual factors, such as sense of coherence and coping style, that mediate or moderate the relationships.^19–21^ Other research, from the sociology tradition, has sought community-level (neighbourhood-level) factors, such as community-level social support. Two well-researched mechanisms that provide such support are social capital and social cohesion.^22^ Social capital and social cohesion have been examined as important protective factors against the development of depression among people at higher risk, such as combat-exposed soldiers,^23^ and more recently among those in post-conflict settings.^24^ Social support has also been reported to have a moderating effect on the symptoms of PTSD among Eritrean and Sudanese asylum seekers in Israel after exposure to trauma.^25^ Social capital and social cohesion have been found to be associated with the health and emotional well-being of Syrian refugee children in Lebanon.^26^ Somasundaram observed, in the context of prolonged conflict and violence (in Sri Lanka), the importance of collective-level action to intervene with social processes and to establish social control and positive family and community relationships.^6^

In the past decade, social science research specifically based on social disorganisation theory has moved beyond structural factors to explore influences of perceived neighbourhood process factors.^27^ This research mainly draws on the seminal work of Sampson and colleagues on neighbourhoods and crime^28^ referring to neighbourhood collective efficacy. Collective efficacy theory improved social disorganisation theory by adding the effects of social cohesion and social control. Collective efficacy was originally defined in Sampson et al's study as the combination of informal social control and social cohesion.^28^ It examines the social interactional relationships among neighbours and whether they care about the common good.^27,28^ The construct of informal social control captures residents’ ability to get together and control negative behaviours in their neighbourhood. Communities with high levels of collective efficacy have significantly lower levels of violence^29^ and burglary.^30^ Communities with higher collective efficacy may promote experiences of safety, calm, optimism and social support.^4^ In such communities, members are more likely to have lower exposure and react more resiliently to chronic adversities, work together to make resources available for rebuilding, and provide mutual support and assistance. Such collective-level factors would have a protective role in the context of exposure to war trauma and daily stressors. The concept of collective efficacy partly overlaps with other social mechanisms, such as social capital.^31^ A distinction between social capital and collective efficacy has been made, where the former is about relationships and the latter is about converting these into action.^32^ However, the literature has not explored the effects of collective action in mitigating the effects of war and conflict on mental health. We contend that collective efficacy buffers and creates resilience to the effects of war trauma and daily stressors on the mental distress of populations exposed to war and conflict.

## The current study: background

In Sri Lanka, civilians in the Northern Province were affected by a 30-year-long armed conflict that ended in May 2009. Previous studies have found high levels of PTSD, anxiety and depression in the population in provinces exposed to this protracted conflict; all were significantly associated with displacement status and past trauma exposure.^33–36^ Qualitative studies in northern Sri Lanka have found that complex mental health and psychosocial problems at the individual, family and community levels in a post-war context impair recovery.^5^ Displacement had been the defining experience of almost all the civilians in the conflict-affected regions in the Northern Province.^37^ Jayawickreme and colleagues identified a plethora of stressors, including common family problems, and found strong correlations between current life stressors and manifestations of traumatic stress resulting in multiple forms of distress.^38^

At the time of the study, there had been a gap of 10 years since the population in the Northern Province had been exposed to a prolonged war and several forced displacements, which provides a unique setting to examine the daily stressors model and specifically the mediation and moderation hypotheses of daily stressors. We chose to examine collective efficacy in this study as one of the major psychosocial consequences of war at the collective level. Subsequent studies will explore other collective measures, such as social capital, trust and social cohesion, drawing on mixed method analysis of both qualitative and quantitative data collected as part of the Office for National Unity and Reconciliation (ONUR) programme. The role of protective factors, specifically informal social control, a core component of collective efficacy in predicting mental health outcomes, has not been examined in this literature. We aimed to examine:

- the role of daily stressors as a mediator and moderator of the association between exposure to war trauma and mental distress, within a socioecological model of distress among internally displaced persons; and
- the protective role of informal social control on the association between exposure to war trauma and mental distress.

## Method

### Study setting and population

This study was carried out in 2019 in the Jaffna, Kilinochchi, Mullaithivu, Vavuniya and Mannar Districts of northern Sri Lanka, the hardest hit by three decades of war and the resettlement process. Within a psychosocial rehabilitation project under the ONUR,^15^ badly affected villages were identified from each of the five districts for assistance after consultation with local and governmental administration working in the area, village leaders and village members (one or more villages are administrated as a *Grama Niladhari* division). Selection of villages was based on high numbers affected by war trauma, female-headed households, poverty, alcoholism, child abuse, domestic violence, school drop-out or irregular attendance, suicide and attempted suicide. The research assistants were university graduates whose first language was Tamil and had completed a social or biological sciences degree with research methods training. They were selected following an aptitude test and interview. They were then trained for 6 months by a team including the first author.

The participants included all adults 18 years or older who were resettled in the village and had experienced war trauma, multiple displacements, injury, detentions, torture, and loss of family, kin, friends, homes, employment and other valued resources. Only Tamil-speaking (forming the overwhelming majority (>95%) in the villages) participants were included. Individuals with severe medical conditions (requiring long-term hospital admissions), epilepsy, psychotic disorders and any psycho-organic conditions (psychiatric disorders due to organic causes, physical diseases) were excluded. Those with any serious illnesses were referred for further treatment. There was a total of 336 participants from the five districts (Jaffna: 69 from Uduththurai village; Killinochchi: 67 from Shanthipuram village; Mullaithivu: 63 from Mallikaiththivu village; Vavuniya: 31 from Lyca and Puliyankulam villages and 36 from Kothandarnochchikulam village; and Mannar: 46 from Ganesapuram village and 24 from Kuruvil village). Information was incomplete for 8 participants.

### Measurements

#### Post-traumatic stress symptoms (PTSS)

Symptoms of PTSD were assessed using the Impact of Event Scale – Revised (IES-R).^39,40^ The IES-R measures the psychological impact of a traumatic event. Its three subscales (Intrusion, Avoidance and Hyperarousal) reflect the degree to which individuals re-experience a traumatic incident, the degree of intrusiveness these re-experiences have for them, any attempts they make to use avoidance/numbing mechanisms in dealing with the consequences of the event, and symptoms of hyperarousal such as anger and irritability, heightened startle response and difficulty concentrating. The items are rated on a 5-point severity scale (1 ‘not at all’, 5 ‘extremely’). The Tamil translation of the scale has been validated in Sri Lanka.^41^ A mean score was generated based on the sum of all endorsed items.

Model fit for factorial validity of the IES-R was tested using confirmatory factor analysis in mPlus (version 8 for Windows) using a weighted least squares means and variance adjusted (WLSMV) estimator. Acceptable model fit was obtained by changing the loading of two items from Intrusion to Hyperarousal (items 2 ‘trouble staying asleep’ and 14 ‘acting or feeling like I was back at that time’) and omitting three items that had unacceptable cross-loadings (items 12 ‘did not deal with them’, 13 ‘my feeling was kind of numb’ and 21 ‘I felt watchful and on guard’). The model fit of the revised measure was χ^2^ = 465.3 (d.f. = 132), *P* < 0.0001; root mean square error of approximation RMSEA = 0.09 (90% CI 0.08–0.10); comparative fit index/Tucker–Lewis Index CFI/TLI = 0.974/0.970 and standardised root mean squared residual SRMR = 0.046. Cronbach's alpha for the revised 18-item measure was 0.952.

#### War trauma inventory

The items constituting the war-related trauma inventory were identified by the ONUR team for the survey based on: previous studies (e.g. Harvard Trauma Questionnaire), our work undertaken to develop an instrument to measure trauma in northern Sri Lanka,^5,36^ initial pilot (qualitative) studies and the team's extensive engagement with populations exposed to the 30-year war and during resettlement. We chose eight items that were typically associated with the war: deaths, arrests, relatives disappearing, relatives abducted, injuries, war-related disabilities, forced recruitment and being a child soldier. From these items, five items based on their univariate relationship to outcomes (depression and PTSS) were selected for exploratory factor analysis using mPlus, as the item responses were dichotomous (yes/no). The selected items were whether the individual had been affected by (a) arrests, (b) missing persons, (c) relatives abducted, (d) injuries and (e) war-related disability. The last item included particularly loss of body part such as a limb or eye (Supplement 1, available at https://doi.org/10.1192/bjo.2023.563). Tests of factorial validity showed loading of items to be from 0.3 to 0.8. Reliability was measured using MacDonald's coefficient omega^42^ given the limitations of Cronbach's alpha unless the items are tau equivalent.^43^ Omega was 0.697.

#### Daily stressors

Four items were selected to measure current daily stressors not directly related to war: (a) ‘Are you satisfied in your family life?’; (b) ‘Do you have sufficient monthly income for your family (from head of your family)?’; (c) ‘Do any of your family members suffer from mental issues?’; (4) ‘Are your children affected physically and/or mentally?’. The last item, including child abuse, was in reference to overt forms of child abuse and exploitation perpetrated by adults. This set of questions were asked broadly, in the context of the period of war and post-war. The responses for the items were dichotomous (yes/no). A sum of the items was used to produce a scale from 0 to 5. Factorial validity was tested for the composite measure using mPlus. Tests of factorial validity showed item loadings to be from 0.48 to 0.93. Reliability as measured using MacDonald's coefficient omega was 0.783.

#### Collective efficacy

We used the ‘informal social control’ subscale of the measure of collective efficacy,^28^ as represented by a five-item Likert-type scale (‘Would you say it is very likely, likely, neither likely nor unlikely, unlikely, or very unlikely?’). Residents were asked about the likelihood that their neighbours could be counted on to intervene in various ways if (a) children were skipping school and hanging out on a street corner; (b) children were spray-painting graffiti on a local building; (c) children were showing disrespect to an adult; (d) a fight broke out in front of their house; and (e) the fire station closest to their home was threatened with budget cuts.^28^ Based on social and cultural relevance, items (b) and (e) were replaced with ‘Children were making noise playing during a *bajan* [religious] session’ and ‘The primary school in the area was to be closed by authorities’ respectively.

The factorial validity of the culturally adapted scale was tested using confirmatory factor analysis. The model fit of the measure was χ^2^ = 88.6 (d.f. 33), *P* < 0.0001; RMSEA = 0.07 (90% CI 0.05–0.09); CFI/TLI = 0.991/0.987 and SRMR =  0.051. Cronbach's alpha for the five-item measure was 0.73.

#### Sociodemographic characteristics

The sociodemographic variables included in the study were gender, age and level of education.

### Data collection

To randomly select participants for the study we used the official lists of resident families in the selected villages, which were kept by the *Grama Niladhari*. Research assistants then approached the selected residence in each village, starting from one residence and then going to the closest next residence in the selected list till the required number of families in each village was recruited. An adult who was literate, willing to answer the questions and present at home at the time of the visit was administered the questionnaire verbally after informed consent was obtained.

The authors assert that all procedures contributing to this work comply with the ethical standards of the relevant national and institutional committees on human experimentation and with the Helsinki Declaration of 1975, as revised in 2008. All procedures involving human participants were approved by the Ethics Review Committee of the Faculty of Medicine, University of Kelaniya, Sri Lanka (approval ERC No.: P/236/11/2019). All participants provided written informed consent to participate in this study.

### Data analysis

Statistical analyses were performed using IBM SPSS (version 24.0 for Windows). We first calculated the descriptive statistics and correlations for the key variables. We then used the SPSS PROCESS macro developed by Hayes^44^ to examine the mediation role of daily stressors in the relationship between exposure to war trauma and PTSS. The macro has been widely used in previous studies to evaluate mediation models using the bias-corrected percentile bootstrap method.^45,46^ All variables were standardised and bootstrap estimates (95% CI) were used to evaluate the theoretical model, based on 5000 bootstrap samples. Previous studies of populations exposed to war trauma have found gender and age differences as confounders in the association between war trauma and psychological outcomes.^9,11^ We included gender and age as covariates in all our analyses.

**Fig. 1:**
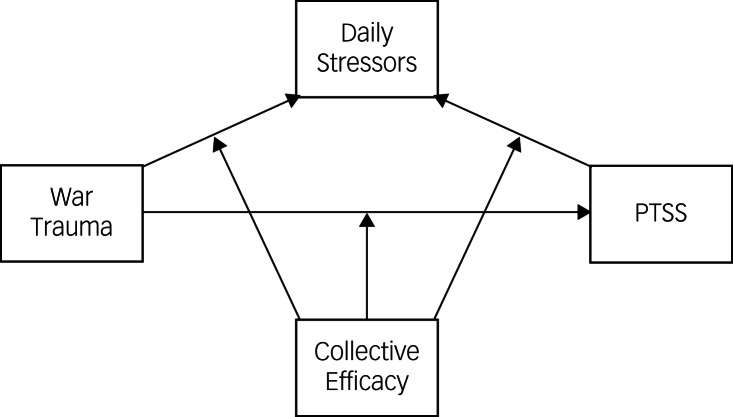
Moderated mediation model for the study.

To examine the direct effect and the mediating effect of daily stressors on the association between exposure to war trauma and PTSS, we used PROCESS Model 4. To test the moderating effect of daily stressors on the association between war trauma and PTSS, we used PROCESS Model 1. The moderated mediation model for the effects of collective efficacy on the relationship between war trauma, daily stressors and PTSS is depicted in Fig. 1. We used PROCESS Model 59 to test this model. To understand the moderating effect more clearly, we conducted separate simple slope analyses for each moderator at low, moderate and high (16th, 50th and 84th percentiles respectively) values. Further details of the justification and analysis steps are provided in Supplements 2 and 3.

## Results

### Preliminary analyses

The key continuous variables in our models, the explanatory variable (war trauma), the dependent variable (PTSS) and the possible mediator and moderator variables (daily stressors and collective efficacy), are significantly correlated (Table 1). The total number of participants was 336. The means, standard deviations and pairwise correlations of all variables are presented in Table 1. Exposure to war trauma was significantly correlated with daily stressors (*r* = 0.22, *P* < 0.01) and PTSS (*r* = 0.13, *P* < 0.05). Daily stressors were in addition significantly related to age (*r* = 0.12, *P* < 0.05) and PTSS (*r* = 0.23, *P* < 0.01). Females had a significantly lower collective efficacy score (*t* = −2.158, *P* < 0.05). There was no difference in the mean PTSS values between the genders (*t* = 0.716, *P* = 0.475). PTSS was significantly related to war trauma (*r* = 0.13, *P* < 0.05) and age (*r* = 0.27, *P* < 0.01).

**Table 1:** Descriptive statistics and correlations among key variables^a^^,^^b^

Variable	*n*	Mean	s.d.	Age	War trauma	Daily stressors	PTSS	Collective efficacy
Age	328	48.22	15.666	1	−0.016	0.118*	0.266**	−0.059
War trauma	315	0.83	0.960	−0.016	1	0.216**	0.130*	0.044
Daily stressors	325	2.55	1.329	0.118*	0.216**	1	0.225**	0.099
PTSS	303	26.74	18.601	0.266**	0.130*	0.225**	1	−0.356**
Collective efficacy	328	15.14	6.453	−0.059	0.044	0.099	−0.356**	1

PTSS, post-traumatic stress symptoms.

a. Total sample size was *n* = 336. Information was incomplete for 8 respondents.

b. Pairwise deletion was used in calculating Pearson's correlation.

*Correlation significant at the 0.05 level (two-tailed); **Correlation significant at the 0.01 level (two-tailed).

### Mediating and moderating effects of daily stressors

The results of the mediation models presented in Table 2 show that the direct effect of war trauma on PTSS was not statistically significant (*B* = 0.11, 95% CI −0.01 to 0.22). After including daily stressors as a mediator, the indirect effect was (*B* = 0.03, 95% CI 0.00–0.06), with both paths, war trauma to daily stressors (*B* = 0.20, 95% CI (0.09–0.31)) and daily stressors to PTSS (*B* = 0.15, 95% CI 0.03–0.26), significant. Age was a significant predictor of PTSS (*B* = 0.23, 95% CI 0.12–0.34) and daily stressors (*B* = 0.17, 95% CI 0.05–0.29). These results show that in the presence of age and gender as covariates, the path from war trauma to PTSS was mediated by daily stressors.

**Table 2:** Summary of mediation results^a^

Outcome	Predictor	*R*	*R*^2^	*F*	*B*	s.e.	95% CI
PTSS		0.34	0.12	8.93***			
	War trauma				0.11	0.06	−0.01 to 0.22
	Daily stressors				0.15*	0.06	0.03 to 0.26
	Age				0.02***	0.00	0.01 to 0.02
	Gender				0.21	0.13	−0.05 to 0.46
Daily stressors		0.26	0.07	6.41***			
	War trauma				0.20***	0.06	0.09 to 0.31
	Age				0.01**	0.00	0.00 to 0.02
	Gender				0.13	0.13	−0.13 to 0.39
Effect		*B*	Bootstrap s.e.	Bootstrap LLCI	Bootstrap ULCI		
Direct	War trauma > PTSS	0.11	0.06	−0.01	0.25		
Indirect	War trauma > daily stressors > PTSS	0.03	0.01	0.00	0.06		

PTSS, post-traumatic stress symptoms; LLCI, lower limit of the confidence interval; ULCI, upper limit of the confidence interval.

a. Bootstrap sample size: 5000.

* *P* < 0.05, **P < 0.01, ***P < 0.001.

Moderation of the relationship between war trauma and PTSS by daily stressors was examined using PROCESS Model 1 (Supplementary Table 1). The results showed that war trauma alone did not significantly predict PTSS (*B* = 0.07, 95% CI −0.05 to 0.18)). However, both daily stressors and the interaction term ‘war trauma × daily stressors’ were significant predictors (*B* = 0.16, 95% CI 0.04–0.27 and *B* = 0.13, 95% CI 0.02–0.25 respectively). The latter result confirms that daily stressors moderate the relationship between war trauma and PTSS.

**Fig. 2:**
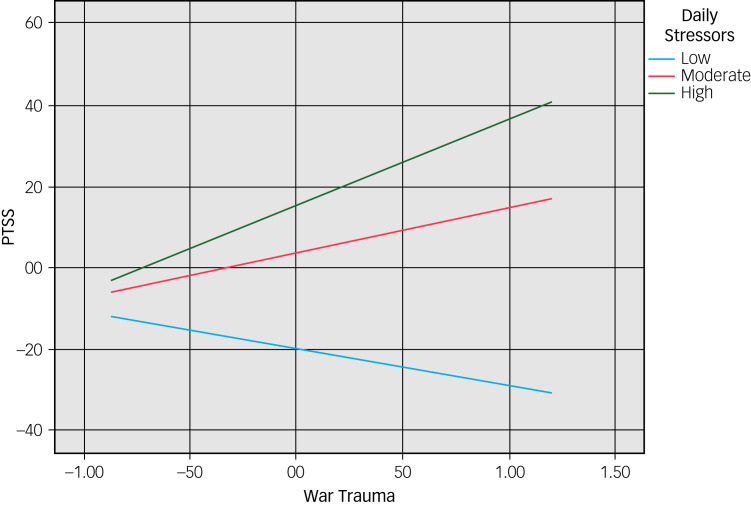
Moderating effect of daily stressors in the relationship between war trauma and post-traumatic stress symptoms (PTSS).

To further examine the moderation, we conducted simple slope analyses. The results (Fig. 2) show that, when the level of daily stressors is low, the predictive effect of war trauma on PTSS is not significant (*b* = −0.09, *t* = −0.86, *P* = 0.39). However, when the level of daily stressors is moderate or high the predictive effect of war trauma on PTSS is positive and significant (*b* = 0.11, *t* = 1.93, *P* = 0.05 and *b* = 0.21, *t* = 2.85, *P* < 0.001 respectively). The slope analyses (Fig. 2) also show that the PTSS level of the ‘high daily stressors’ group is higher than that of the ‘moderate daily stressors’ group, indicating that higher daily stressors increase the predictive effect of war trauma on PTSS.

### Mediating and moderating effects of collective efficacy

We used the multiple mediator model (PROCESS model 4) (Supplementary Fig 3) to test whether collective efficacy is a mediator of the association between war trauma and PTSS. We found (Supplementary Table 2) that collective efficacy was not a mediator of the association between war trauma and PTSS in the presence of daily stressors.

The moderated mediation model was used to assess whether collective efficacy moderated the direct effect of war trauma on PTSS and the indirect effect of daily stressors in the mediation model. The results (Table 3) show that the interaction term ‘war trauma × collective efficacy’ is *B* = 0.11, 95% CI = 0.00–0.24. The model shows that moderation by collective efficacy is conditional on its level: the direct effects are significant only at high levels (1.06, 84th percentile), whereas the indirect effects are significant at both moderate (−0.02, 50th percentile) and high levels (1.06, 84th percentile).

**Table 3:** Moderated mediation model: collective efficacy as moderator with daily stressors as mediator

Outcome	Predictors						
		*R*	*R*^2^	*F*	*B*	s.e.	95% CI
PTSS		0.52	0.27	14.02***			
	Age				0.23***	0.06	0.12 to 0.34
	Gender				0.08	0.05	−0.03 to 0.18
	War trauma				0.07	0.05	−0.04 to 0.18
	Daily stressors				0.20***	0.06	0.09 to 0.31
	Collective efficacy				−0.39	0.06	−0.50 to −0.28
	War trauma × collective efficacy				0.11	0.06	0.00 to 0.24
	Daily stressors × collective efficacy				0.02	0.05	−0.09 to 0.12
Effect	Collective efficacy values	*B*	Bootstrap s.e.	Bootstrap LLCI	Bootstrap ULCI		
War trauma > PTSS							
	−1.26	−0.07	0.10	−0.27	0.12		
Direct	−0.02	0.07	0.06	−0.0.04	0.17		
	1.06	0.19	0.08	0.04	0.34		
	−1.26	0.03	0.03	−0.01	0.10		
Indirect	−0.02	0.04	0.02	0.01	0.07		
	1.06	0.04	0.02	0.00	0.09		

PTSS, post-traumatic stress symptoms; LLCI, lower limit of the confidence interval; ULCI, upper limit of the confidence interval.

a. Bootstrap sample size: 5000.

*P < 0.05, **P < 0.01, ***P < 0.001.

**Fig. 3:**
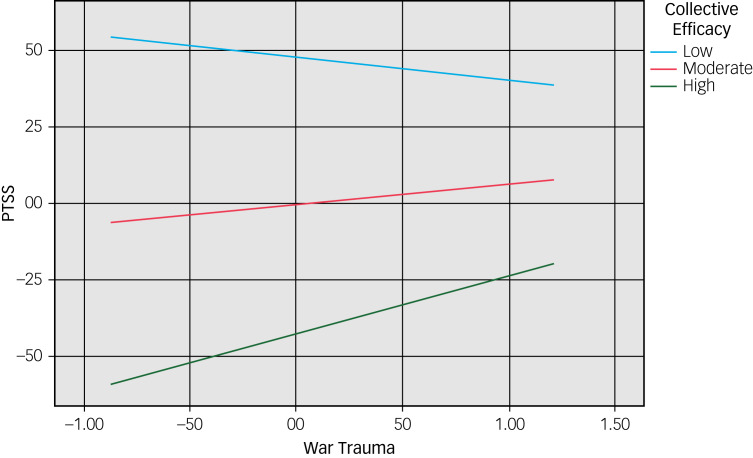
Moderating effect of collective efficacy in the relation between war trauma and post-traumatic stress symptoms (PTSS).

This moderated association is shown in the results of the simple slope analysis (Fig. 3). The slope at a high level of collective efficacy is positive and significant (*b* = 0.19, *t* = 2.45, *P* = 0.01), showing a positive relationship between war trauma and PTSS. However, the two slopes at low (*t* = −0.73, *P* = 0.17) and moderate levels (*t* = 1.20, *P* = 0.23) (16th and 50th percentiles respectively) were not significant. The simple slopes also illustrate that at higher levels of collective efficacy, the level of PTSS at a given score of war trauma is lower, indicating a protective effect of collective efficacy.

In addition, the test of conditional indirect effects (Table 3) shows that the indirect association between war trauma and PTSS through daily stressors was significant at moderate and high levels (50th and 84th percentiles) of collective efficacy, but was non-significant at low levels (16th percentile) of collective efficacy. This indicates that at higher levels of collective efficacy, the mediating role of daily stressors in the relationship between war trauma and PTSS was stronger.

## Discussion

This study tested the well-known model of daily stressors affecting post-traumatic distress^8^ in a post-conflict context among a population of internally displaced persons in Sri Lanka 10 years after their exposure to war. We found that current daily stressors fully mediated the association between war trauma and psychological distress. Similar results have been reported among internally displaced persons in Nepal^12^ and Sierra Leone.^13^ The respondents in our study were internally displaced persons who were resettled mostly in their areas of origin and the measure of daily stressors consisted of chronic family and environmental stressors and not items of potentially traumatic experiences (such as childhood abuse or intimate partner violence). The strong mediation effect of these chronic, low-intensity social, material and mental stressors found in our study may be due to the depletion of family resources over decades of war and lack of development over a decade post-war in these settings.^5^

We tested the moderating effect of these daily stressors on the relationship between past exposure to war trauma and current psychological distress. Our results show that, when daily stressors are moderate or high, there is a significant relationship between past war trauma and psychological distress. One explanation is that when internally displaced persons are under higher levels of stress due to burden of daily living, their recollections of past exposure to war trauma have a stronger influence on PTSS. Actions to reduce daily stressors may lessen the intensity of war-related trauma and facilitate the process of healing, congruent with recommendations given for refugees by Miller & Rasmussen.^15^

We selected a well-accepted community level factor, collective efficacy,^28^ and tested its effects on both daily stressors and the relationship between war trauma and mental distress. We found that collective efficacy moderated the effect of daily stressors within the daily stressors mediation model and that it is a significant moderator only at high levels of collective efficacy. This is congruent with the findings of Ursano et al,^47^ where lower levels of PTSD were found in communities with higher levels of collective efficacy, following exposure to hurricane Katrina in New Orleans. To our understanding, this is the first study to find this protective effect among forcibly displaced persons.

The concept of collective efficacy partly overlaps with other social mechanisms, such as social capital.^31^ However, there are nuanced and important differences. According to Cagney & Wen, ‘social capital is about relationships and collective efficacy is about converting those relationships into action’.^32^ Collective efficacy also has a social cohesion component and an action component.^48,49^ Our measure of ‘informal social control’ included items on actions a community would take. The study findings provide initial evidence that enhancing collective efficacy through informal social control could be an additional strategy to deliver effective mental health and psychosocial support (MHPSS) programmes among internally displaced persons. Future studies need to test the efficacy of such interventions in settings with prolonged distress in post-conflict situations.

Somasundaram et al highlight that in Sri Lanka, the traditional social structures and cultural practices, such as the caste system and religious rituals, play a vital role in shaping collective efficacy. In addition, it is essential to consider the importance of social harmony, religious beliefs and community traditions when studying collective efficacy in this region.^50,51^

### Implications and future research

The findings from this study have practical and programmatic relevance for MHPSS interventions that would also reduce ongoing daily stressors for internally displaced persons not only in Sri Lanka but in other communities in similar contexts around the world. In the case of Sri Lanka, these findings endorse programmes that have been designed to address wider psychosocial support services for interventions for individual and family trauma.^6^ The Inter-Agency Standing Committee (IASC) recommends that after addressing basic needs such as food, shelter, security and basic healthcare, an MHPSS response in participatory, safe and socially appropriate ways that protects local people's dignity, strengthens local social supports and mobilises community networks should be implemented.^52^ In a cultural context, the functioning family is the basic building block and foundation of Tamil communities, and it would be essential for trained community workers to promote the restoration of functioning family units. They could work with families to help them trace missing members, participate in cultural grieving ceremonies for the dead, improve relationships and correct misunderstandings among members, re-establish hierarchical responsibilities, create income-generating opportunities for the family and generally encourage unity and positive dynamics. A positive sense of collective efficacy, where people see their actions having good results, leads to a sense of agency, acting proactively as a community for their own common good. In the wider ONUR psychosocial rehabilitation programme for the war-affected communities in northern Sri Lanka and in other similar programmes, individual-, family- and community-level interventions that included reducing daily stressors were not only effective for individuals and families but also showed improvements in community-level functioning that, in turn, had positive effects on individual and family functioning.^5–7,18^

We suggest that future research should further explore the unique ways collective efficacy manifests and contributes to resilience in north-eastern Sri Lanka. Furthermore, research should examine how collective efficacy may be influenced by the ongoing reconciliation and post-conflict reconstruction processes in the country.^5,7^

### Limitations

There are several limitations in our study that need to be considered. These results are not representative of the districts, as the selection of villages was biased towards those most severely affected by the war. However, a random sample of households was selected in each village. The selection of the respondents was influenced by the presence of adults at home at the time of the visit by data collection teams, which might explain the higher proportion of females in the sample. The relatively modest sample size afforded limited statistical power to detect potentially larger effects. The selection of the circumscribed set of items for war trauma and daily stressors based on their robust psychometric profiles precluded the use of the broader set of items. The informal social control applied in our study was a measure of this construct at the individual level owing to the nature of the sample in the study. Ursano et al^47^ in their study following a hurricane in New Orleans found collective efficacy to be associated with significantly lower PTSD symptoms as an individual-level perception and a community-level capacity. Although we examined relationships between war trauma, daily stressors and psychological distress, the use of cross-sectional data precludes causal inferences.

## Supporting information

Somasundaram et al. supplementary material Somasundaram et al. supplementary material

## Data Availability

The data that support the findings of this study are available from the corresponding author (D.S.) on reasonable request.
