# Supplementary material for: Effect of daily stressors and collective efficacy on post-traumatic stress symptoms among internally displaced persons in post-war northern Sri Lanka
Source: BJPsych Open. 2023 Oct 11;9(6):e180. doi: 10.1192/bjo.2023.563 (PMC10594160; doi:10.1192/bjo.2023.563)
Supplement: Somasundaram et al. supplementary material [file S205647242300563Xsup001.docx]

Supplement 1 : Justification for selection and analysis of trauma events for the study

The larger ONUR study collected a wide range of information at baseline, as it was designed for an MHPSS intervention. For the purposes of the analysis for this study, we based our item selection on the widely applied daily stressor models (Miller and Rasmussen 2018). As these events predated the survey by ten years, we decided to include only historically significant events with the highest degree of salience and impact on the participants. This selection process considered existing instruments, such as the Harvard Trauma Questionnaire, as well as previous research conducted by the authors. We chose eight items that were distinctively related to war-related events. The items included: Deaths, Arrests, Relatives disappearing, Relatives abducted, Injuries, War related disabilities, Forced recruitment, and Child soldiers.

For the analysis, we subjected the eight items to a selection process to ensure reasonable construct validity and reliability, allowing them to be used as a composite for analyses. We tested their univariate relationships with two mental health outcomes, PTSD and Depression. To further refine our selection, we conducted an Exploratory Factor Analysis, with the binary items analyzed using tetrachoric correlations in mPlus.

Rasmussen et al.(2018) have raised the issue that checklist items are better considered as causative (formative) indicators. While they offer suggestions for addressing this issue, there is currently no consensus within the MHPSS literature on adopting these methods. Consequently, we adhered to the standard procedures for validating and testing the reliability of measures employed in latent variable analyses. We acknowledge the need for future research and for the field to reach a consensus on the most suitable measurement of war trauma and other traumatic events.

Supplement 2: Justification of methods of mediation and moderation analysis

Preamble

Mediation and moderation analyses are two techniques that allow to address questions of why and when variables are related. Baron and Kenny (1986) made the distinction that, mediation as the “mechanism” through which a focal independent variable is able to influence the variable of interest and a moderator is a variable that affects the direction and strength of the relation between an independent or predictor variable and a dependant or criterion variable.

The integration of both into a single model provides researchers ability to examine even more nuanced relationships between variables. Hayes and Preacher (2013) refer to them as moderated mediation or conditional process models that allow for researchers to test if direct and indirect effects are moderated by another variable. This allows an understanding of the boundary conditions under which associations between two (or more) variables occur (Aguinis, 2004)

Selection and sequence of models tested.

STEP 1: We commenced with a well-accepted model based on the “Daily stressors” theory that has been tested in many post conflict and refugee situations. In this study we tested the “daily stressor model’ , when there was long duration between exposure and outcome, as at the time of data collection, the final war that most participants were exposed to was 10 years ago (year 2009). We postulated that (i) the association between war trauma (WT) and psychological distress (PTSS) would be mediated by current daily stressors; and (ii) that the association between WT and PTSS would be moderated by the strength of daily stressors.

We used PROCESS model 4 , to test the former, the mediation model ( see diagram S1) and PROCESS model 1 , to test the latter , the moderation model ( see diagram S2)

Figure S1

*Daily Stressors*

*WT*

*PTSS*

Model 4

Figure S1

Figure S2

Model 1

*Daily Stressors*

*WT*

*PTSS*

STEP 2: Based on our research and relevant literature (see introduction), we postulated that individuals exposed to war trauma would perceive that, Collective Efficacy (CE) is a protective factor for the development of psychological distress.

We used the multiple mediator model (PROCESS Model 4) (Figure S3), to test whether CE, is a mediator of the association between WT and PTSS. We found (see results below) that CE was not a mediator of the association between WT and PTSS, in the presence of DS.

Figure S3 Multiple Mediator model

War Trauma

PTSS

Figure S3

Based on prior literature (see introduction) and our own field experience of protective effects of CE , we tested whether CE moderated the relationships in the daily stressor model ( ie: between, WT and DS; DS and PTSS ; and WT and PTSS). To test all three paths, we used PROCESS model 59 (figure S4 and figure 1 in manuscript).

Figure S4: Moderated Mediation model for the study

Daily Stressors

Collective Efficacy

War Trauma

PTSS

Supplement 3 : Additional results not presented in manuscript.

Model 4 : Moderation model to test moderation of association between WT and PTSS, by DS.

Table S1 Moderated Model: Daily Stressors (DS) as moderator of association between War Trauma (WT) and Psychological distress (PTSS)

| Outcome (Y) | Predictors (x) |  |  |  |  |  |  |
| --- | --- | --- | --- | --- | --- | --- | --- |
|  |  | R | R^2^ | F | B | SE | 95%CI |
| PTSS |  | 0.36 | 0.13 | 8.25*** |  |  |  |
|  | Age |  |  |  | **0.26***** | 0.06 | **0.14-0.37** |
|  | Sex |  |  |  | 0.10 | 0.06 | -0.01-0.22 |
|  | War Trauma (WT) |  |  |  | 0.07 | 0.06 | -0.05-0.18 |
|  | Daily Stressors (DS) |  |  |  | **0.16**** | 0.06 | **0.04- 0.27** |
|  | WT X DS |  |  |  | **0.13*** | 0.06 | **0.02- 0.25** |
| Effect | DS Values | B | Boot SE | Boot LLCI | Boot ULCI |  |  |
| WT >PTSS |  |  |  |  |  |  |  |
|  | -1.17 | -0.09 | 0.11 | -0.30 | 0.12 |  |  |
|  | 0.34 | **0.11*** | 0,06 | **0.00** | **0.22** |  |  |
|  | 1.09 | **0.21**** | 0.07 | **0.07** | **0.36** |  |  |

Bootstrap sample size = 5000. *CI* confidence interval, *LL* low limit, *UL* upper limit

* *p* < .05, ***p* < .01, ****p* < .001

Model 5 : Multiple mediator model to test mediation by DS and CE

Table S2 Multiple Mediator Model: Daily Stressors (DS) and Collective Efficacy (CE) as mediators of association between War Trauma (WT) and Psychological distress (PTSS)

| Outcome (Y) | Predictors (x) |  |  |  |  |  |  |
| --- | --- | --- | --- | --- | --- | --- | --- |
|  |  | R | R^2^ | F | B | SE | 95%CI |
| PTSS |  | 0.51 | 0.26 | 18.69*** |  |  |  |
|  | War Trauma (WT) |  |  |  | 0.09 | 0.05 | -0.02-0.19 |
|  | Daily Stressors (DS) |  |  |  | **0.20*** | 0.06 | **0.09 –0.31** |
|  | Collective Efficacy (CE) |  |  |  | -0.40 | 0.05 | -0.51-(-0.29) |
|  | Age |  |  |  | **0.23***** | 0.05 | **0.12-0.33** |
|  | Sex |  |  |  | 0.07 | 0.05 | -0.04-0.17 |
| DS |  | 0.24 | 0.06 | 5.23*** |  |  |  |
|  | War Trauma (WT) |  |  |  | **0.18***** | 0.06 | **0.06-0.30** |
|  | Age |  |  |  | 0.16** | 0.06 | 0.04- 0.27 |
|  | Sex |  |  |  | 0.06 | 0.06 | -0.06-0.18 |
| CE |  | 0.10 | 0.01 | 0.88 |  |  |  |
|  | War Trauma (WT) |  |  |  | 0.01 | 0.06 | -0.11-0.13 |
|  | Age |  |  |  | -0.08 | 0.06 | -0.20-0.04 |
|  | Sex |  |  |  | -0.07 | 0.06 | -0.19-0.05 |
| Effect |  | B | Boot SE | Boot LLCI | Boot ULCI |  |  |
|  |  |  |  |  |  |  |  |
| Direct | WT > PTSS | 0.09 | 0.05 | -0.02 | 0.19 |  |  |
| Indirect | WT>DS>PTSS | **0.04** | 0.01 | **0.01** | **0.07** |  |  |
| Indirect | WT>CE>PTSS | 0.00 | 0.02 | -0.05 | 0.04 |  |  |

Bootstrap sample size = 5000. *CI* confidence interval, *LL* low limit, *UL* upper limit

* *p* < .05, ***p* < .01, ****p* < .001

**References**

1 Baron RM, Kenny DA. The moderator–mediator variable distinction in social psychological research: Conceptual, strategic, and statistical considerations. *Journal of Personality and Social Psychology* 1986; **51**: 1173–82.

2 Hayes AF, Preacher KJ. Conditional process modeling: Using structural equation modeling to examine contingent causal processes. In *Structural equation modeling: A second course, 2nd ed.*: 219–66. IAP Information Age Publishing, 2013.

3 Aguinis H. *Regression analysis for categorical moderators*. Guilford Press, 2004.
